# Which virtual education methods do e‑students prefer? Design and validation of Virtual Education Preferences Questionnaire (VEPQ)

**DOI:** 10.1186/s12909-023-04687-2

**Published:** 2023-10-03

**Authors:** Zahra Karimian, Asieh Barkhor, Manoosh Mehrabi, Laleh Khojasteh

**Affiliations:** 1 Department of e-Learning in Medical Sciences, Virtual School and Center of Excellence in e-Learning, Shiraz University of Medical Sciences, Shiraz, Iran; 2 Virtual School and Center of Excellence in e-Learning, Shiraz University of Medical Sciences, Shiraz, Iran; 3 Department of English Language, School of Paramedical Sciences, Shiraz University of Medical Sciences, Shiraz, Iran

**Keywords:** Educational preferences, Virtual education, Validation, Student, e-learning

## Abstract

**Background:**

With the prevalence of new technologies and evolving student learning styles, virtual teaching methods have become increasingly popular. As a result, more and more students are opting to learn online. However, one common concern is that they may feel disconnected from their teachers, leading to feelings of loneliness and doubts about the quality of education they are receiving. To address this issue, a study was conducted to gather data on students' preferences for virtual education and to validate a tool for measuring students' preferences for virtual education.

**Methods:**

The research was conducted in a mixed method with a quantitative–qualitative sequence. A virtual education preferences questionnaire (VEPQ) for students with a total of 17 items was created and validated as part of the qualitative component by looking at the theoretical underpinnings and experts' opinions in the focus group. The scale of the six-point Likert questionnaire was from very high to very low. To validate the tool and determine preferences, exploratory factor analysis was used. A total of 155 samples answered the questions and the data were analyzed using SPSS-24 software.

**Results:**

A total of 155 complete questionnaires were returned; among them, 110 (71%) were filled out by women, 73 (47.1%) by respondents between the ages of 36 and 45, 107 (69%) were already employed in paramedical fields, and 48 (31%) were enrolled in a medical school. The opinions of ten experts were used to confirm the face validity of the questionnaire. With CVI = 0.924 and CVR = 0.805, content validity was verified. Using the internal consistency method of the questions with a Cronbach's alpha coefficient of R = 0.824, the validity of the entire questionnaire was confirmed. Exploratory factor analysis revealed that a total of five components—self-directed projects (29.58%), e-content (13.00%), online presentation (10.97%), face-to-face interactions (9.12%), and text interactions (7.11%) had the highest load, with a total of 69.77% of the structure explaining virtual education preferences. The factor analysis test and the suitability of the sample are both confirmed by the value of KMO = 0.721 and the significance of Pvalue < 0.001.

**Conclusion:**

It appears that the highly valid tool developed can be used to ascertain the educational preferences of students. Also, the high factor load of self-directed educational methods and e-content shows that independence and flexibility in time and place are more important for students.

## Introduction

Students learn in different ways and have different educational preferences [1], in part because universities today have a very diverse student body in terms of age range, experiences, culture, level of preparation, and learning styles. This diversity makes it harder than ever for university professors to inspire students and advance their understanding [2]. Furthermore, the level of student satisfaction with the curriculum and learning environment is another challenge facing professors today [3], and the teaching style and interaction between the professors and students are two of the most significant and influencing factors in students' academic engagement [4]. The choice of teaching style marks the pivotal point in this influence, demonstrating why university professors should carefully consider their teaching and training strategies [5, 6]. Norman postulates that how information is presented has an impact on how well learners learn [7]. The existence of a contradiction between the subject matter of learning and the methods of instruction, inBertolami's opinion, is one of the primary causes of students' despair and hopelessness about the curriculum [8]. Even with the best teachers, some students may still struggle to learn because they have different learning preferences [9], which means they prefer to think, analyze, and acquire and process imagination [10]. They also prefer to see and hear information in different ways. The results of research in this area have been varied. While some students prefer practical methods, others prefer teaching strategies that utilize visual aids [3, 11, 12].

New teaching and learning techniques are constantly being added thanks to the development of new technology. The gradual adoption of new techniques in recent decades in the education of students includes virtual education delivered both online and offline, the use of multimedia, etc. By enabling the use of network technology to create, expedite, provide, and facilitate education at any time and place [13], virtual education has created a new type of learning environment. It is a form of education delivery through electronic devices and includes using computers or other electronic devices in a variety of ways to initiate the learning process or create educational materials [14].

The unique experience of using three visual, auditory, and textual learning methods simultaneously is a feature of virtual education [15]. With the active participation of the learner, virtual education shifts learning from a teacher-centered and pamphlet-writing method to one that is learner-centered. It is possible to give each student more time to learn and review the material in this way. Additionally, it allows people to access information without regard to their location, and from an economic perspective, it is efficient because it gives professors more time to conduct research and educate more students. With this method of instruction, there will not be any physical restrictions on learning, and the student can take advantage of learning at any time or place [16].

In summary, virtual or online learning is defined as the use of electronic technology and media to deliver, support, and enhance learning and teaching, involving communication between educators and learners utilizing online content [17]. This method of learning can provide students with easier and more effective access to a wider range and greater amount of information and can help them develop self-directed learning skills [18]. Although virtual learning and teaching are now being combined with traditional face-to-face instruction in many fields, it is particularly important to pay attention to creating suitable learning environments for students studying in virtual fields. This is because their access to teachers, physical resources, and face-to-face interaction is limited, which can make them feel isolated from the learning environment and possibly receive less timely feedback or have difficulty expressing their requests and preferences compared to other students.

In the fields of medical sciences, the use and application of new technologies are very advanced [19, 20], and many fields are even presented virtually. Many people choose to take virtual courses in order to overcome time and space constraints. On the other hand, virtual students may experience loneliness or worry about getting a sufficient and complete education due to the lack of access to teachers and face-to-face interaction.

Therefore, it is crucial to understand this group of students' preferences in terms of education. Although there is much research on the preferred instructional strategies in face-to-face classes, there are a few on the preferred instructional strategies in online courses. Therefore, it is very effective and useful to identify these preferences when planning educational programs. The objective of this research was to identify the virtual education techniques favored by online learners and to validate a tool for measuring students' preferences for virtual education.

## Methodology

### Design and method

The goal of the current study was to build and validate a tool to identify and measure the educational preferences of students in virtual fields. It was designed using a mixed qualitative and quantitative methodology. In the qualitative phase of the research, seven faculty members and educational experts with expertise in medical education and e-learning participated in a focus group to identify the descriptive elements of educational preferences. In the quantitative phase, a survey method was used to implement the tool developed with a validation approach on Shiraz University of Medical Sciences' virtual students in 2020–2021, and the construct validity analysis method was used to assess the tool's validity.

### Population and statistical sample

Seven medical education and e-learning experts were carefully chosen as the statistical samples for the qualitative section. These professionals were Shiraz University of Medical Sciences virtual course instructors, who had a minimum of five years of teaching experience in the field of online courses.

In the quantitative phase of the research, a virtual population of Shiraz University of Medical Sciences postgraduate students who were pursuing studies in virtual fields made up the statistical population. The four courses at Shiraz University of Medical Sciences—Master of Medical Education, Master of E-Learning, Master of Community-based Education, and MPH of Health Policymaking —had a combined enrollment of 300 students for the 2020–2021 academic year. Various viewpoints exist regarding the appropriate sample size for exploratory factor analysis research. At least five times as many variables (items) are approved by Hatcher 1994 [21], and some experts advise at least ten [22]. At least 51 samples, according to Lawley and Maxwell, are more than the required number of variables in short questionnaires [23]. For our research, seven to ten times sample questions were needed, given that the original questionnaire had 17 questions. As a result, the number of samples was estimated to be between 119 and 170, but 180 subjects received questionnaires due to the possibility of non-return questionnaires.

### Inclusion and exclusion criteria

All Shiraz University of Medical Sciences master's degree students in virtual fields who gave informed consent to participate in the study were included, and those who did not respond to more than 20% of the questions were excluded.

### Tools and methods of data collection

To investigate the "virtual learning preferences" items from the perspective of students, we first reviewed relevant articles. Although some similar studies had addressed this topic [24, 25], we needed to examine the learning preferences of students who had experienced virtual education programs at our university. Therefore, it was necessary to develop a tool that was comprehensive and appropriate. For this reason, we first identified questionnaire items in the qualitative phase of the research.

During the qualitative phase, we conducted a focus group and held a meeting with seven experts present. The experts included two individuals with Ph. D.s in e-learning, one with an MD/Ph.D. in medical education, two experts in health education, two academic researchers specializing in medical education and e-learning, and group coordinators holding MSc degrees in educational technology. Brainstorming was used to list common methods in student education. The participants were purposefully selected among individuals who were faculty members and representatives of each virtual field of the university and were familiar with the teaching methods used in their respective educational groups.

The focus group was conducted in a 4-h session divided into two parts: the first part lasted for 120 min, during which the researchers expressed the work objectives and provided necessary explanations about various e-learning methods. Participants were asked to list all the teaching methods used in the virtual fields of the university. Initially, all participants recorded the methods on a piece of paper, and after collecting the papers, in the open discussion section, participants talked about the commonly used methods, and additional items were added to the list. After a break and refreshments (30 min), in the second part (90 min), the items were written on the whiteboard, similar items were merged, and duplicates were removed. Finally, the list of e-teaching methods was extracted and confirmed by consensus. Notably, there are many different methods of e-teaching based on the theoretical foundations, but the initial items of the questionnaire were considered based on the methods used by the professors of Shiraz University of Medical Sciences in the related field and the items that were not used in professors’ teaching methods of the course were eliminated from the list.

In the quantitative phase, a Virtual Education Preferences Questionnaire (VEPQ) with 20 items and a 6-point Likert scale from very high = 6 to very low = 1 was designed. The questionnaire was distributed randomly and collected face-to-face from students in the university's virtual fields.

### Data analysis

In the qualitative phase, the brainstorming items collected during the focus group meeting were listed, and a list of 20 items was compiled by summarizing the data. During the face validity assessment, three items were removed based on feedback received. Ultimately, only 17 items were considered suitable and included in the final questionnaire. These 17 items were then used in the exploratory factor analysis. Following the distribution and collection of data in the quantitative phase and the examination of the opinions of virtual students, these data were analyzed and categorized using exploratory factor analysis. In the factor analysis, the KMO index was used to determine the adequacy of the sample before the data were classified into classes based on the explained sharing.

**Fig. 1:**
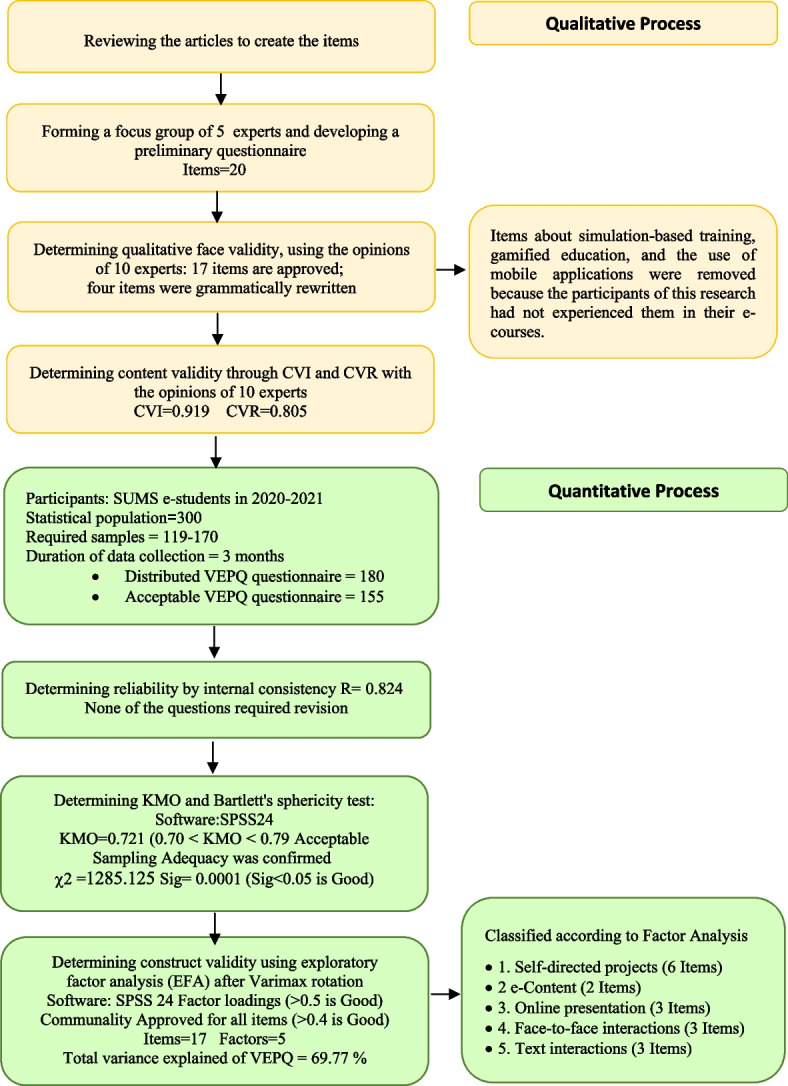
Research steps in developing the Virtual Education Preferences Questionnaire (VEPQ)

All research steps in developing the VEPQ are shown in Fig. 1 at a glance.

### Ethical considerations

The students gave their informed consent before filling out the surveys in an anonymous manner. The Research Vice-Chancellor of Shiraz University of Medical Sciences approved this project as a component of the e-learning master's thesis, and the Shiraz University of Medical Sciences Ethics Committee approved it in accordance with the code of ethics IR.SUMS.REC.1398.413.

## Results

Based on the descriptive findings of demographic characteristics, 155 completed questionnaires were returned; 110 (71%) of the participants were female, 73 (47.1%) were in the age range of 36 to 45 years, 107 (69%) had prior paramedical education, and 48 (31%) had prior medical education. In Table 1, the specifics of the demographic traits are displayed (Table 1).

**Table 1:** Demographic characteristics of students participating in the research

Characteristics	Sub-categories	Frequency	
		**N**	%
Gender	• Male	45	29
	• Female	110	71
Age (Years)	• 22–35	55	35.5
	• 36–45	73	47.1
	• > 46	27	17.4
	• Mean	38.13 ± 8.29	
Marital status	• Single	41	26.5
	• Married	114	73.5
Previous field of study	• Medicine	48	31
	• Para-medicine	107	69
The current field of study	• MSc-E-Learning in Medical Sciences	27	17.4
	• MSc-Medical Education	61	39.4
	• MPH- Health Policymaking	43	27.7
	• MSc-Community based education	24	15.5
Employment status	• Faculty members	8	5.2
	• Experts	95	61.3
	• Self-employment	33	21.3
	• Unemployed	19	12.3

Given that this was the first time the VEPQ questionnaire had been used, it was essential to test the tool's psychometric properties. By polling 7 experts in e-learning and medical education on 20 key items, the VEPQ psychometric was developed. Then, from the perspective of ten experts, face validity and content validity were examined. Educational management (2), e-learning (3), MD. Ph.D. of medical education (4), and educational technology experts (1) were chosen. None of the individuals who participated in the face and content validity assessment were part of the initial focus group.

### Face validity

Ten experts were sent the initial questionnaire and asked to provide qualitative feedback on the face validity of the items. By reviewing the comments, they determined that the content of the three questions was inappropriate. There were items about training through simulation methods and virtual reality, but these methods were not used in the training of the students who participated in this study, so these questions were eliminated. Additionally, 4 questions had to have their grammar fixed, and finally, a questionnaire with 17 questions was created to be assessed for its content validity.

### Content validity

* The CVI and CVR indices of a questionnaire with 17 questions were calculated to assess the content validity of the questionnaire. The CVI index is presented by Waltz and Bausell and consists of three sub-indices, ranging from 1 to 4, for relevance, clarity, and simplicity. In the data analysis, items that received a score of 3 or 4 are acceptable, as is the average score of more than 79% [26].

* The CVR index categorizes the necessity of each questionnaire item into three categories: necessary, useful but not necessary, and unnecessary. In the analysis of items, an item is deemed acceptable if it achieves the necessity index, and the average score is then calculated using the formula below.

$$CVR=\frac{{n}_{e}-{}^{N}\!\left/ \!{}_{2}\right.}{{}^{N}\!\left/ \!{}_{2}\right.}$$

In this formula, N represents the total number of experts, and ne represents the number of experts who selected the required option. According to this method provided by Lawshe, a minimum score of 0.625 is expected if ten experts have commented on the items [27].

### Reliability

Cronbach's alpha index was used to examine the internal consistency of each item to determine the items' reliability; a score of more than 80 percent is an excellent level of item reliability. Then, by selecting the ‘If deleted item’ option, the reliability of each item was calculated based on the number of items in the questionnaire; in this method, if deleting an item results in an increase in reliability, the question should be examined. In addition, the dependability of each area was examined. Table 2 displays the results for CVI and CVR content validity and reliability (Table 2).

**Table 2:** Psychometric properties of content validity and reliability of the VEPQ

Components	Items	Content Validity				Reliability	
		**CVR**		**CVI**		**Cronbach's Alpha**	
		Essential	Simple	Clear	Relevant	Factors	If Item Deleted
Text interactions	Q01	90	100	100	100	0.806	0.808
	Q02	70	80	90	100		0.802
	Q03	100	80	80	90		0.824
Online presentation	Q04	100	80	90	100	0.675	0.810
	Q05	80	90	100	90		0.807
	Q06	100	80	80	100		0.819
e-Content	Q07	80	100	90	100	0.525	0.821
	Q08	80	80	80	70		0.829
Self-Directed Projects	Q09	100	100	100	100	0.854	0.816
	Q10	80	90	100	100		0.811
	Q11	80	100	100	100		0.804
	Q12	70	90	90	100		0.810
	Q13	70	80	90	100		0.809
	Q14	70	100	100	90		0.808
Face-to-face interactions	Q15	60	90	100	100	0.709	0.820
	Q16	60	90	100	100		0.826
	Q17	80	70	80	80		0.824
Total	*	*	0.953	0.924	0.882	-	-
		CVR_Total_ = 0.805	CVI _Total_ = 0.919			R = 0.824	

### Construct validity

Given that this instrument was created for the first time to identify factors and construct validity, exploratory factor analysis was employed. Before and after factor analysis, it is necessary to examine the following criteria to determine the construct validity of the questionnaire:

#### Criteria before determining the factors

Before factor analysis and determining the components of the questionnaire, it is necessary to conduct the suitability test using the KMO test and Bartlett's test of sphericity. These two tests confirm the adequacy of the sample size and the appropriateness of the test. The appropriateness of the factor analysis test is confirmed by the value of KMO = 0.721 and the significance of Pvalue < 0.001 as shown in Table 3.

**Table 3:** The results of KMO and Bartlett's Test of VEPQ

Kaiser–Meyer–Olkin Measure of Sampling Adequacy		0.721
0 < KMO<0.49 Unacceptable	0.50<Kmo <0.59 Weak	0.60<KMO<0.69 Medium
0.70<KMO<0.79 Acceptable	0.80<KMO<0.89 Appropriate	0.90<KMO<1.00 Excellent
Bartlett's Test of Sphericity	Approx. Chi-Square	1285.125
	df	136
	Sig	< 0.0001

#### Criteria after determining factors

After ensuring that the factor analysis test is appropriate for the research and that it has been implemented, it is necessary to check the appropriate variables to be kept in the research. The suitability of the variables is assessed for this purpose using metrics like factor load and sharing rate. A value higher than 0.5 is considered an acceptable factor load, and values higher than 0.4 are considered acceptable for keeping a variable in the study. As can be seen from Table 4, every question on the survey has been reported with a participation rate of at least 0.5, meaning that every question is acceptable (Table 4).

**Table 4:** The commonalities of VEPQ based on exploratory analysis

Questions	Extraction	Questions	Extraction
Q1	0.777	Q10	0.608
Q2	0.827	Q11	0.817
Q3	0.650	Q12	0.776
Q4	0.548	Q13	0.721
Q5	0.749	Q14	0.688
Q6	0.498	Q15	0.684
Q7	0.679	Q16	0.832
Q8	0.701	Q17	0.747
Q9	0.560		

The reduction of variables and classification of factors is the main objective of factor analysis. When making the initial prediction, we considered six factors. However, using exploratory factor analysis and rotating the factors according to the "Kaiser" criteria, 5 factors were extracted, and two questions were incorporated into other areas. Only factors whose squared factor load, or "eigenvalue," is greater than one are accepted according to the "Kaiser" criterion. Table 5 lists the number of factors and their corresponding values (Table 5).

**Table 5:** Total variance explained of VEPQ based on exploratory analysis

Components	Initial Eigenvalues			Extraction Sums of Squared Loadings			Rotation Sums of Squared Loadings		
	Total	% of Variance	Cumulative %	Total	% of Variance	Cumulative %	Total	% of Variance	Cumulative %
1	5.03	29.58	29.58	5.03	29.58	29.58	3.65	21.46	21.46
2	2.21	13.00	42.58	2.21	13.00	42.58	2.74	16.09	37.55
3	1.87	10.97	53.55	1.87	10.97	53.55	2.06	12.09	49.64
4	1.55	9.12	62.67	1.55	9.12	62.67	1.95	11.46	61.10
5	1.21	7.11	69.77	1.21	7.11	**69.77**	1.48	8.68	69.77
6	0.93								
7	0.73								

**Fig. 2:**
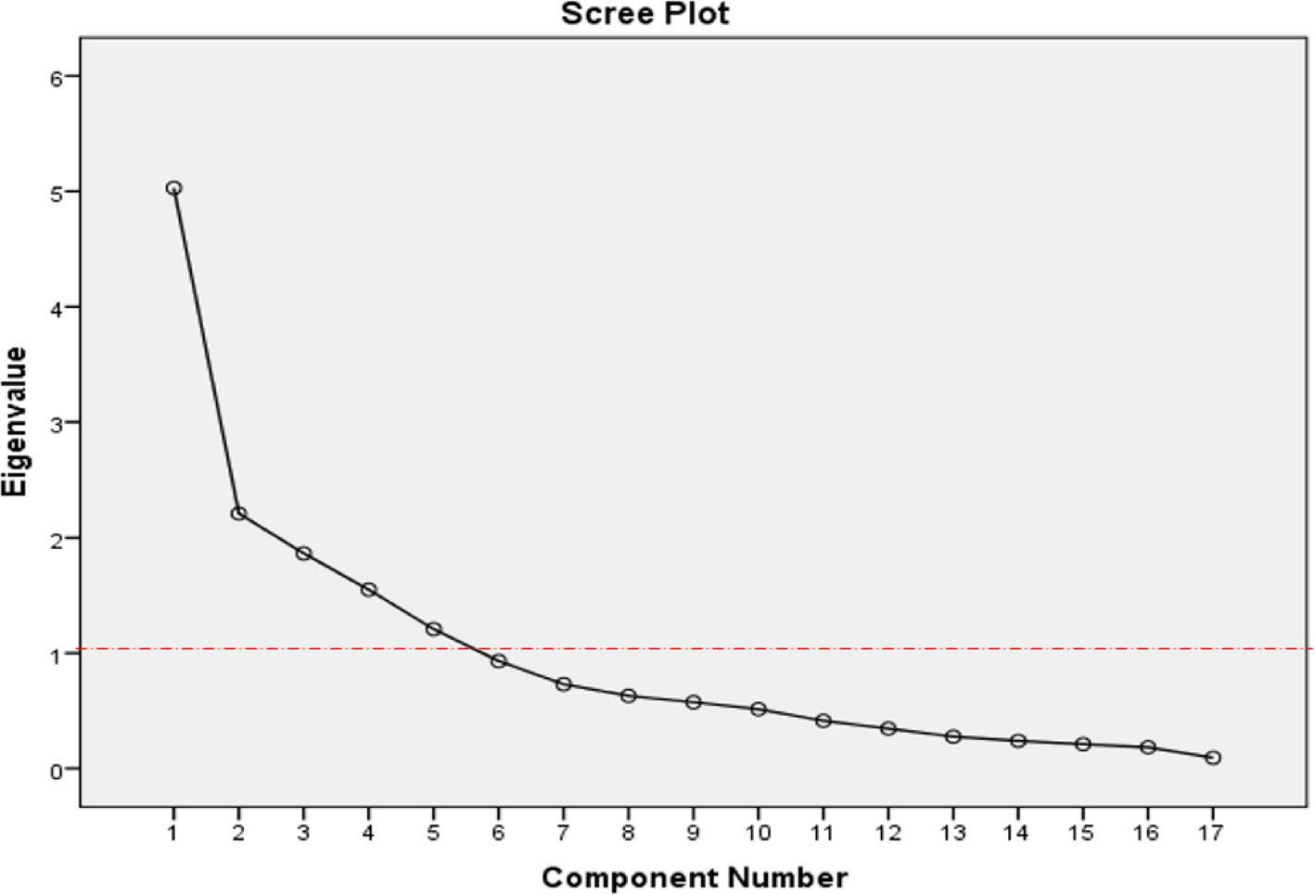
Screen plot of VEPQ based on exploratory factor analysis

Five retrieved components account for 69.77% of the variation of the VEPQ, according to the findings. Seventeen items were divided into 5 groups based on varimax rotation, as shown in Table 6. Also included is a scree plot (Fig. 2). The first factor's wide distance from the other factors suggests that it has the largest factor load of all the factors (29.58%), whereas the other factors have closer factor loads (Fig. 2).

**Table 6:** Varimax rotated component matrix of VEPQ based on exploratory analysis

Components	Items	Factor load order				
		1	2	3	4	5
Self-Directed Projects	• Q9. I prefer to read and translate scientific texts as part of my homework	.628				
	• Q10. I learn better when I do a practical project (e-Content and App development, etc.)	.633				
	• Q11. Writing a scientific article is more effective and informative in my learning	.820				
	• Q12. Analyzing, criticizing, and evaluating articles or different situations is an effective method	.860				
	• Q13. I learn better in assignments that are based on planning, problem-solving, and presenting a solution	.747				
	• Q14 I prefer to review and integrate several articles/models and create a new article/model	.774				
e-Content	• Q7. I prefer to have multimedia and educational videos before the class		.781			
	• Q8. I prefer to have the handout of the course (PDF handout)		.821			
Online presentation	• Q4. I learn better when the professor teaches online in a virtual class			.513		
	• Q5. I learn better when my classmates present the webinars and conferences			.757		
	• Q6. I prefer questions & answers in the virtual class			.667		
Face-to-face interactions	• Q15. It is necessary to hold face-to-face meetings at the beginning of the semester for introduction and clarification				.755	
	• Q16. I prefer to have some intensive face-to-face meetings during the semester to provide a summary				.898	
	• Q17. I prefer to participate in face-to-face troubleshooting sessions before the exams				.665	
Text interactions	• Q1. Discussions boards and forum environments increase my ability to criticize and analyze					.845
	• Q2. feel that I learn better by sharing content and ideas through Wiki and the forum					.805
	• Q3. I prefer to ask my questions in text form in online environments					.777

Extraction Method: Principal Component Analysis. Rotation Method: Varimax with Kaiser Normalization

According to the Rotated Component Matrix table's findings, the extracted components with the factor load explained 69.77% of the construction of virtual education preferences. These components were extracted in the following order of importance: self-directed projects (6 items), e-content (2 items), simultaneous online presentations (3 items), and face-to-face training (3 items).

Also, Table 7 shows the correlation matrix between the items of the questionnaire. The level of correlation between factors is categorized into four groups: 0 to 0.1, 0.1 to 0.3, 0.3 to 0.5, and > 0.5. Table 7 shows that each item correlates with at least 0.1 of the other items of the questionnaire. Although the correlation between items is mostly moderate, the adequacy of the sample and the suitability of the factor analysis are confirmed by other indices such as KMO and Bartlett's test of sphericity (Table 7).

**Table 7:**
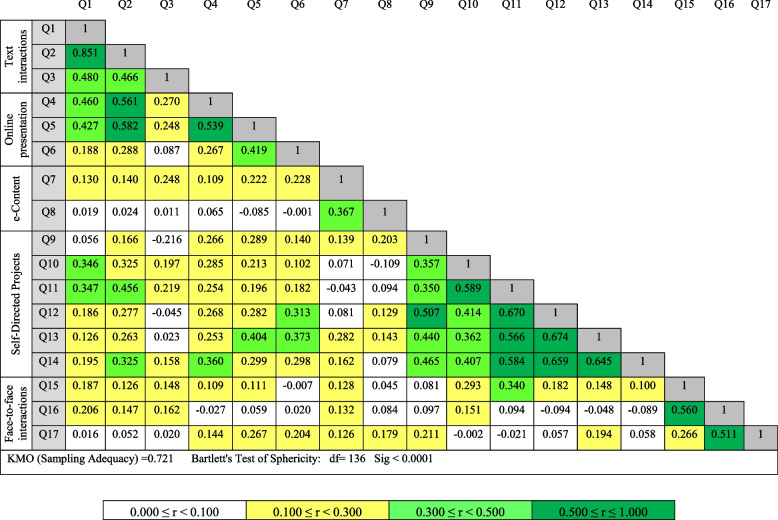
Correlation matrix after varimax rotation

## Discussion

### Content validity

According to the findings of the content validity analysis performed using the CVI and CVR methods, the results indicated that the content validity had been appropriately validated. By polling 10 experts, it is predicted that there will be at least 62 percent agreement based on the Lawshe model [27]. The total average CVR was 0.805, which is a very good value, and the majority of the items have values between 0.7 and 1; only in the case of two items, the value of CVR = 60 which is still acceptable, noting that their CVI has been confirmed as excellent (= 1). CVI was also used to confirm validity. Waltz and Bausell provided this CVI index. The experts were asked to rate each item on a scale of 1 to 4 for relevance, clarity, and simplicity to determine the CVI. We divided the number of respondents who selected options 3 and 4 by the total number of experts to arrive at the index. Although the result between 0.7 and 0.79 can be re-examined and corrected [26], it is acceptable if the result is greater than 0.79. Based on the findings of the research in Table 2, the average of the entire questionnaire was obtained at CVI = 0.919, which is a very excellent and reliable value, and in the case of individual items, the values were often more than 80%, and in a few cases, it was about 0.7.

### Reliability

Additionally, the reliability values support the questionnaire's acceptability in terms of the questions' internal consistency. One of the most popular methods for assessing a question's internal consistency and reliability, particularly in surveys using a Likert scale, is Cronbach's alpha test [28, 29]. It implies that it has a more thorough internal analysis of the questionnaire's dimensions [29–31] and that the higher the correlation between the questions, the higher the Cronbach's alpha value will be [32]. A value between 0.8 and 0.9 indicates excellent reliability and a reliability score of at least 0.70 is required [33, 34].

Also, in the ‘Deleted If Item’ mode of the SPSS software, each item was deleted in order and the reliability of the rest of the items was calculated. If the reliability value increases by removing an item, it means that the item has a problem and it may be interpretable that we can correct or remove the option. As seen in the reliability results of the questionnaire, by removing each item, the reliability value of the rest of the questions decreased, and this indicates that the items had a positive effect on the overall reliability. The Cronbach's alpha value of the questionnaire with 17 questions was 0.824, which is at an excellent level. Of course, it should be noted that the questionnaire had 17 questions. This number is acceptable because Cronbach's alpha can be reduced more when there are fewer questions. In Cronbach's alpha analysis, it is important to keep in mind that the number of questions is also a factor; the fewer the questions, the lower the Cronbach's alpha [34, 35]. Cronbach's alpha is affected by both the internal correlation of the questions and their number. It is probably expected that as the number of questions in this questionnaire rises, so will the reliability value.

### Construct validity

Based on the results, the five extracted factors account for 69.77% of the variance in students' virtual education preferences. The magnitude of this variance and the conformity of the findings to the hypothesis suggest that the derived criteria are validly used. The significance of Bartlett's sphericity test and the value of KMO both show that the sample is adequate. KMO must be between 0.70 and 0.79 for the sample to be considered adequate. The obtained KMO value (0.721) demonstrates that the sample count is within acceptable bounds. The appropriateness of the test is also confirmed by the value of Sig obtained in Bartlett's sphericity test [36, 37] (Table 3).

Table 3 (Commonalities of VEPQ items) shows that each of the variables explained more than about 0.6 of the sharing. A value > 0.6 is an appropriate index because the minimum expected for this index is 0.5 [38] and sometimes 0.4 and 0.3 are, also acceptable in some articles [39].

### Factor loading analysis

Based on the results obtained from factor analysis, questionnaire items were categorized into 5 general components and 17 items. In total, the extracted 5 components explain 69.77%, that is, about 70% of the concept of educational preferences, and it shows that the questionnaire has been able to a large extent to define the virtual education preferences from the students' point of view. Notably, we included preferences in the questionnaire that the students experienced in the teaching methods of their professors. Since the studied fields were interdisciplinary in nature and close to behavioral sciences, some teaching methods such as simulators, virtual reality, augmented reality, and game creation were used less and students did not have any experience regarding them. Therefore, in future research and considering the nature of the field, it is necessary to add additional items to the questionnaire. The components of the questionnaire are listed below in order of priority:

#### Self-directed projects

The first and most important component from the point of view of virtual students was self-directed projects. Table 5 explains about 29.58% of the total variance. In other words, the students' top priorities were learning through hands-on projects and taking responsibility for their own homework. Given that the majority of the students were women, employed, married, and of an average age for adults, they preferred independent learning. The theories of adult education [40] and self-directed learning are applicable to this finding [41, 42]. This can be influenced by adults’ prior experience in addition to age. According to Thompson's research, adults’ preferences for education are more influenced by their prior work experience than by their age [43].

#### E-content

E-content, the second element of virtual education preferences, accounts for 13% of the overall variance. The majority of research highlights the value of pre-prepared e-content, which is highly preferred due to its adaptability to time and place as well as the needs of students [44, 45]. Another consideration is that multimedia e-content enhances learning by engaging different sensory channels [46], is better suited to students' learning preferences, and gives them a more enjoyable learning experience [47]. Of course, other studies, like those of Fincher, have revealed that older people favor traditional methods and have less interest in education related to technology [48].

Of course, other research such as Fincher has shown that older people prefer traditional methods and have less desire for technology-related education [48]. The fact that our study participants are virtual students—most of whom are women—who juggle work, family, and other commitments that make it difficult for them to travel to and participate in in-person classes may account for this discrepancy.

#### Online presentation

The third component, which accounted for 10.97 percent of the overall variance, is from the perspective of the students taking synchronous online courses. Although the learner's independence was given more weight in the first two priorities, students still prefer to interact with their professors in order to solve problems and continue their face-to-face interactions with them. The adaptability of online methods has been highlighted in many research studies [49–51], but synchronous methods can be crucial in ensuring that ambiguities are clarified and questions are raised, as well as learning from peers in the classroom. Walker thinks that the ability of professors to provide immediate feedback to students, engage in an interactive dialogue with them, and answer their questions in real time is very important for effective teaching [51].

#### Face-to-face interactions

Face-to-face training, the fourth factor, accounts for about 9.12% of the overall variance. Real-time interaction with instructors ranks fourth on the importance scale, closely behind online presentations although many of the students in the virtual fields studied here are married with children. This is in contrast with other research that reported that older people prefer traditional face-to-face methods compared to electronic methods [48].

This may be because people who work and study simultaneously value flexibility and the ability to adapt to changing circumstances more than they value making arbitrary decisions. The first two preferences (self-directed projects and multimedia electronic content) are the most adaptable to specific circumstances because they are not time- or location-dependent. The face-to-face troubleshooting sessions that take place prior to the exams have the highest factor load of all the face-to-face training topics. Oncu and Cakir contend that people prefer face-to-face instruction because, despite its time flexibility, online communication can be less effective for testing and problem-solving due to little interaction and quick feedback [52].

#### Text interactions

Text interactions were ranked as the fifth priority from the perspective of the students (7.11 percent). This component focuses more on synchronous and asynchronous textual interactions, which are used when people would rather interact without regard to place or time constraints. In general, students prefer to communicate via text platforms [53], but the relative importance of this component to other components reveals that, although asynchronous text methods like forums or wikis promote collaboration with peers and information gathering [53, 54], these tools have limitations because it is difficult to provide prompt feedback and facilitate smooth discussion [54]. Students may find it less appealing because they cannot comprehend emotions or nonverbal language through textual expressions. According to research, timely feedback significantly affects learning, but asynchronous methods are less likely to offer this feature. Additionally, multimedia methods may be better suited to students' learning preferences. Students favor multimedia methods over text methods based on multimedia principles [46].

## Conclusion

In this research, we developed and validated a tool for measuring students' educational preferences, and based on the findings, the priority of students' educational preferences was extracted.

The indicators obtained from the measurement of reliability, content validity, and construct validity showed that this tool has the necessary quality to measure students' educational preferences and has good validity. Also According to the research's findings, it appears that students in virtual fields prefer independent methods and are less reliant on their environment, which can be largely attributed to the circumstances of their personal and professional lives. Project-based learning techniques and online content were also preferred by students who were married, in the workforce, and women with both professional and personal obligations. However, as a supplement to learning, synchronous online and in-person methods can maintain the interaction between professors and students. The hybrid approach is therefore a better choice for students. The results of the tool validation study indicated that it can be a reliable tool for gauging students' educational preferences due to its content validity, construct validity, and reliability.

### Strengths and limitations

#### Strength

Previous research has considered the educational preferences of students, but with the rise of virtual education and an increasing number of students studying in virtual fields, less attention has been given to the preferences of this group. This tool could be useful for future research in this area. The tool has been reviewed by experts in both qualitative and quantitative aspects, analyzed using statistical methods, and deemed acceptable based on the indicators obtained.

#### Limitation

The validity indicators adequately support the fact that this questionnaire was created for the first time, but given the study's one-time nature and recommendations for future research, it has an inherent limitation, so its validity should be checked once more with a different population. Additionally, since the current research is based on the virtual education methods that students who had experience in virtual fields had used, it excluded techniques like gamification, application, simulation, and virtual and augmented reality. Therefore, other e-learning strategies should be researched as well. In our survey, the e-content category had only two items within its subset. However, some sources suggest that at least three items are needed in each subset for structural validity analysis or factor analysis. The results of this study were obtained through exploratory factor analysis. To achieve better validity, future studies could increase the sample size or add new teaching methods, thus potentially increasing the number of items in this component.

### Recommendation for future studies

As this tool was being developed for the first time, we utilized exploratory factor analysis. For future research, we recommend employing confirmatory factor analysis with larger sample sizes. It is important to note that the e-learning methods examined in our research were based on interdisciplinary fields like medical education, e-learning in medical sciences, and community-based education. Therefore, depending on the participants' field of study, additional educational methods may need to be included in the questionnaire.

## Data Availability

The datasets used and/or analysed during the current study are available from the corresponding author on reasonable request.

## Abbreviations

VEPQ: Virtual Education Preferences Questionnaire
SUMS: Shiraz University of Medical Sciences
CVR: Content Validity Ratio
CVI: Content Validity Index

## References

[1] Samarakoon L, Fernando T, Rodrigo C (2013). Learning styles and approaches to learning among medical undergraduates and postgraduates. BMC Med Educ.

[2] Meehan-Andrews TA (2009). Teaching mode efficiency and learning preferences of first year nursing students. Nurse Educ Today.

[3] Murphy RJ, Gray SA, Straja SR, Bogert MC (2004). Student learning preferences and teaching implications. J Dent Educ.

[4] Hafen CA, Ruzek EA, Gregory A, Allen JP, Mikami AY (2015). Focusing on teacher-student interactions eliminates the negative impact of students' disruptive behavior on teacher perceptions. Int J Behav Dev.

[5] Shuster GF, Learn CD, Duncan R (2003). A strategy for involving on-campus and distance students in a nursing research course. J Contin Educ Nurs.

[6] Mafyan F, Nouhi E, Abbaszadeh A (2014). Effect of blended electronic education on learning and self-efficiency in nursing students in the cardiovascular intensive care courses. JNE.

[7] Norman G. When will learning style go out of style?. Adv in Health Sci Educ. 2009;14:1–4. 10.1007/s10459-009-9155-5. 10.1007/s10459-009-9155-5 19189223

[8] Bertolami CN (2001). Rationalizing the dental curriculum in light of current disease prevalence and patient demand for treatment: form vs. content. J Dent Educ.

[9] Al-Roomy MA (2023). The Relationship Among Students' Learning Styles, Health Sciences Colleges, and Grade Point Average (GPA). Adv Med Educ Pract..

[10] Mills DW (2002). Applying what we know: Student learning styles. Retrieved April.

[11] Dickinson KJ, Bass BL, Graviss EA, Nguyen DT, Pei KY (2021). How learning preferences and teaching styles influence effectiveness of surgical educators. Am J Surg.

[12] Al Maghraby MA, Alshami AM. Learning style and teaching method preferences of Saudi students of physical therapy. J Family Community Med. 2013;20(3):192–7. 10.4103/2230-8229.122017. 10.4103/2230-8229.122017 PMC3957174 24672278

[13] Esichaikul V, Myint Aung W, Bechter C, Rehman M (2013). Development and evaluation of wiki collaboration space for e-Learning. J Enterp Inf Manag.

[14] Mayer RE (2003). Elements of a science of e-learning. J Educ Comput Res.

[15] Saberi A, Kazempour E, Porkar A (2018). Feasibility of Utilizing Virtual Education from the viewpoints of Professors, Students and Information technology Staff (IT) in Guilan University of Medical Sciences. RME.

[16] Molanapour R (2006). Step by Step of e-Learning for Student.

[17] Howlett D, Vincent T, Gainsborough N, Fairclough J, Taylor N, Cohen J (2009). Integration of a case-based online module into an undergraduate curriculum: What is involved and is it effective?. Elearn Digit Med.

[18] Saiyad S, Virk A, Mahajan R, Singh T (2020). Online teaching in medical training: Establishing good online teaching practices from cumulative experience. Int J Appl Basic Med Res.

[19] Bediang G, Stoll B, Geissbuhler A, Klohn AM, Stuckelberger A, Nko'o S, Chastonay P (2013). Computer literacy and e-learning perception in Cameroon: the case of Yaounde Faculty of Medicine and Biomedical Sciences. BMC Med Educ.

[20] Choules AP (2007). The use of elearning in medical education: a review of the current situation. Postgrad Med J.

[21] Hatcher  L (1994). A Step-by-Step Approach to Using the SAS® System for Factor Analysis and Structural Equation Modeling.

[22] Nunnally, J.C. An Overview of Psychological Measurement. In: Wolman, B.B. (eds) Clinical Diagnosis of Mental Disorders. 1978. Springer, Boston, MA. 10.1007/978-1-4684-2490-4_4.

[23] Lawley DN, Maxwell AE (1971). Factor analysis as a statistical method.

[24] Keeling Ch, Haugestad A (2020). Digital student preferences: a study of blended learning in Norwegian higher education. Nordic J Mod Lang Methodol.

[25] Magalong SJM, Prudente M. Development and Validation of Next Generation Blended Learning Environment Questionnaire for Senior High School Students. IC4E '20: Proceedings of the 2020 11th International Conference on E-Education, E-Business, E-Management, and E-Learning, January 2020, Pages 213–217 10.1145/3377571.3379434).

[26] Waltz CF, Bausell BR. Nursing research: design statistics and computer analysis. Davis FA. 1981.

[27] Lawshe CH (1975). A quantitative approach to content validity. Pers Psychol.

[28] Streiner D (2003). Starting at the beginning: an introduction to coefficient alpha and internal consistency. J Pers Assess.

[29] Cronbach LJ (1951). Coefficient alpha and the internal structure of tests. Psychometrika.

[30] Leontitsis A, Pagge J (2007). A simulation approach on Cronbach’s alpha statistical significance. Math Comput Simul.

[31] Gliem JA, Gliem RR. Calculating, interpreting, and reporting Cronbach’s alpha reliability coefficient for Likert-type scales. Midwest Research-to-Practice Conference in Adult, Continuing, and Community Education. 2003. Retrieved from.

[32] Tavakol M, Dennick R (2011). Making sense of Cronbach's alpha. Int J Med Educ.

[33] Mohammadbeigi A, Mohammadsalehi N, Aligol M (2015). Validity and Reliability of the Instruments and Types of MeasurmentS in Health Applied Researches. JRUMS.

[34] DeVellis RF. Scale development: Theory and applications. Sage Publications. 2011.

[35] Nunnally J, Bernstein L (1994). Psychometric theory.

[36] Thomson RG, De Brún A, Flynn D, et al. Factors that influence variation in clinical decision-making about thrombolysis in the treatment of acute ischaemic stroke: results of a discrete choice experiment. Southampton (UK): NIHR Journals Library; 2017 Jan. (Health Services and Delivery Research, No. 5.4.) Appendix 5, Factor analysis of Institutional Culture Scale. Available from: https://www.ncbi.nlm.nih.gov/books/NBK410188/. 28151613

[37] Comrey AL, Lee HB. A First Course in Factor Analysis. Psychology Press. 2013.

[38] Arash H, Maryam A (2017). Structral Equation Modeling and Factor Analysis.

[39] Samuels, P. (2017) Advice on Exploratory Factor Analysis. Technical Report. ResearchGate, 9/06/2017. Official: https://www.open-access.bcu.ac.uk/6076/.

[40] Mukhalalati BA, Taylor A (2019). Adult learning theories in context: a quick guide for healthcare professional educators. J Med Educ Curric Dev.

[41] Palis AG, Quiros PA (2014). Adult learning principles and presentation pearls. Middle East Afr J Ophthalmol.

[42] Arogundade RA (2011). Adult learning principles for effective teaching in Radiology programmes: A review of the literature. West Afr J Med.

[43] Thompson C, Sheckley BG (1997). Differences in classroom teaching preferences between traditional and adult BSN students. J Nurs Educ.

[44] Khojasteh L, Karimian Z, Farahmandi AY (2022). E-content development of English language courses during COVID-19: a comprehensive analysis of students’ satisfaction. J Comput Educ.

[45] Zalat MM, Hamed MS, Bolbol SA (2021). The experiences, challenges, and acceptance of e-learning as a tool for teaching during the COVID-19 pandemic among university medical staff. PLoS One..

[46] Mayer RE (2008). Applying the science of learning: evidence-based principles for the design of multimedia instruction. Am Psychol.

[47] Issa N, Schuller M, Santacaterina S, Shapiro M, Wang E, Mayer RE, DaRosa DA (2011). Applying multimedia design principles enhances learning in medical education. Med Educ.

[48] Fischer SH, David D, Crotty BH, Dierks M, Safran C (2014). Acceptance and use of health information technology by community-dwelling elders. Int J Med Inform.

[49] Yekefallah L, Namdar P, Panahi R, Dehghankar L (2021). Factors related to students' satisfaction with holding e-learning during the Covid-19 pandemic based on the dimensions of e-learning. Heliyon.

[50] Veletsianos G, Houlden S (2020). Radical flexibility and relationality as responses to education in times of crisis. Postdigit Sci Educ.

[51] Walker SL, Fraser BJ (2005). Development and validation of an instrument for assessing distance education learning environments in higher education: the distance education learning environments survey (DELES) Learn. Environ Res.

[52] Oncu S, Cakir H (2011). Research in online learning environments: Priorities and methodologies. Comput Educ.

[53] Ferrante JM, Friedman A, Shaw EK, Howard J, Cohen DJ, Shahidi L (2016). Lessons learned designing and using an online discussion forum for care coordinators in primary care. Qual Health Res.

[54] Wilkerson JM, Iantaffi A, Grey JA, Bockting WO, Rosser BRS (2014). Recommendations for internet-based qualitative health research with hard-to-reach populations. Qual Health Res.

